# Cryogenic 3D Printing of Super Soft Hydrogels

**DOI:** 10.1038/s41598-017-16668-9

**Published:** 2017-11-24

**Authors:** Zhengchu Tan, Cristian Parisi, Lucy Di Silvio, Daniele Dini, Antonio Elia Forte

**Affiliations:** 1 Department of Mechanical Engineering, Imperial College London, South Kensington Campus, Exhibition Road, London, SW7 2AZ United Kingdom; 2 Tissue Engineering and Biophotonics Division, King’s College London, Guy’s Hospital, Great Maze Pond, London, SE1 9RT United Kingdom; 3 Department of Bioengineering, Imperial College London, South Kensington Campus, Exhibition Road, London, SW7 2AZ United Kingdom

## Abstract

Conventional 3D bioprinting allows fabrication of 3D scaffolds for biomedical applications. In this contribution we present a cryogenic 3D printing method able to produce stable 3D structures by utilising the liquid to solid phase change of a composite hydrogel (CH) ink. This is achieved by rapidly cooling the ink solution below its freezing point using solid carbon dioxide (CO_2_) in an isopropanol bath. The setup was able to successfully create 3D complex geometrical structures, with an average compressive stiffness of O(1) kPa (0.49 ± 0.04 kPa stress at 30% compressive strain) and therefore mimics the mechanical properties of the softest tissues found in the human body (e.g. brain and lung). The method was further validated by showing that the 3D printed material was well matched to the cast-moulded equivalent in terms of mechanical properties and microstructure. A preliminary biological evaluation on the 3D printed material, coated with collagen type I, poly-L-lysine and gelatine, was performed by seeding human dermal fibroblasts. Cells showed good attachment and viability on the collagen-coated 3D printed CH. This greatly widens the range of applications for the cryogenically 3D printed CH structures, from soft tissue phantoms for surgical training and simulations to mechanobiology and tissue engineering.

## Introduction

In the past three decades, 3D bioprinting has become one of the leading techniques for the replication of real tissue geometries, with the potential to mimic the soft tissue microstructure. Hence, bioprinting is currently the focus of several rapidly developing research fields. Recent applications include printing full human organs to contribute towards the shortage of organ donors^1^. With the development of new soft tissue materials that can be used as printing inks, the field of biological 3D printing has grown exponentially, giving rise to the extrusion of living cells suspended in the printing ink^2–6^. A wide range of bioinks used for 3D printing tissue scaffolds have been summarized in a recent review carried out by and Munaz *et al*.^7^, although there is no quantification of stiffness past the qualitative distinction between soft and hard tissues.

It has been shown that the stiffness of the majority of human tissues lies within the order of a few kPa^8^. Furthermore, in specific cases, cell differentiation and regeneration is promoted in tissue scaffolds that exhibit mechanical properties similar to those of the real tissue^9–13^. Therefore, a 3D printing technique that is able to produce geometrically and mechanically accurate scaffolds could hold enormous potential in regenerative medicine and biomimetics^14^. This reinforces the importance of soft 3D printing. To the best of our knowledge, there is a lack of studies focusing on bioprinting very soft materials with stiffness O(1) kPa. One of the causes of this is the inability of extremely soft materials to withstand their own weight: the printed structure is usually too soft to hold its shape or allow further layers to be built on top of it. A few methods that have been developed by other researchers are reported here.

Hinton *et al*.^15^ have developed a technique for free-form extrusion-based 3D printing of biological structures (e.g. arterial branches) using alginate, collagen and fibrin gels as printing inks and a gelatine slurry as a support bath^15^. The technique was able to achieve a resolution of ~200 µm demonstrated through the printing of a scaled down human brain using an alginate bioink. However, the stiffness of the alginate ink was reported to be O(10) kPa, and therefore not comparable with that of super soft tissues, such as human brain or lung (O(1) kPa^15–18^). In another study, Lozano *et al*.^19^ used a RGD modified gellan gum 1 wt% hydrogel bioink with encapsulated cortical neuron cells. The authors were able to demonstrate the ability to print soft 3D cell-laden constructs. However, the printing process was achieved through a hand-held device, hence lacking precision, and the material stiffness was not characterised.

Adamkiewicz *et al*.^20^ introduced a novel cryogenic 3D printing method using liquid nitrogen. The conceptual idea behind the cryogenic method is that it allows inks in a solution state to transform into a solid state, thus allowing stable structures to be built in 3D using a layer-by-layer approach, without the need for a support bath. However, the stiffness of the hydrogel ink was not reported and the precision of the printing method was not discussed^20^. The cryogenic method was also used to create 2D constructs for implants by Wang *et al*.^21^, who utilised a substrate cooled by coolant flow to create the cryogenic stage. Again, mechanical characterisation of the printed structure was not reported.

Therefore, this article demonstrates the fabrication of mechanically accurate 3D printed composite hydrogels that mimic the stiffness of super soft tissues through the use of a novel printing setup based on cryogenic theory. Solid carbon dioxide (dry ice) and an isopropanol thermal conductive bath was used to achieve the cyrogenic stage, which is a safer alternative to liquid nitrogen. The ink used in this work is a composite hydrogel of poly(vinyl) alcohol (PVA) and Phytagel, which has been pioneered by Leibinger *et al*.^22^ and Forte *et al*.^23–25^ to mimic soft tissues, such as brain, with stiffness of O(1) kPa.

A further advantage of this novel 3D printing technique over traditional cast moulding methods resides in the possibility to produce hollow structures of super soft hydrogels. Interconnected holes make soft hollow structures impossible to extract from a mould using traditional cast moulding techniques.

The aims of the study are as follows: (i) to provide mechanical evidence showing the 3D printed material mimics real brain tissue, providing the same response as the casted material^23^, through unconfined compression tests, (ii) to demonstrate the capabilities of this printing technique by achieving hollow 3D printed structures, whose continuity through the layers has also been assessed using Scanning Electron Microscopy (SEM) analysis, and (iii) to evaluate the viability of cells in direct contact with the printed material in order to confirm the potential for future mechanobiological studies.

## Results

### Cryogenic 3D Printing Technique

The resolution achieved by the cryogenic printing method was approximately 262 µm at 2 ml/h flow rate, 5 mm/s print speed. This is demonstrated in Section 2.3 where details of the microstructure of the samples are investigated using SEM. This resolution was comparable to values found in literature by Hinton and co-workers who report a resolution ~200 µm^15^.

### Printed vs. Cast mechanical properties and comparison with Brain Tissue

**Figure 1:**
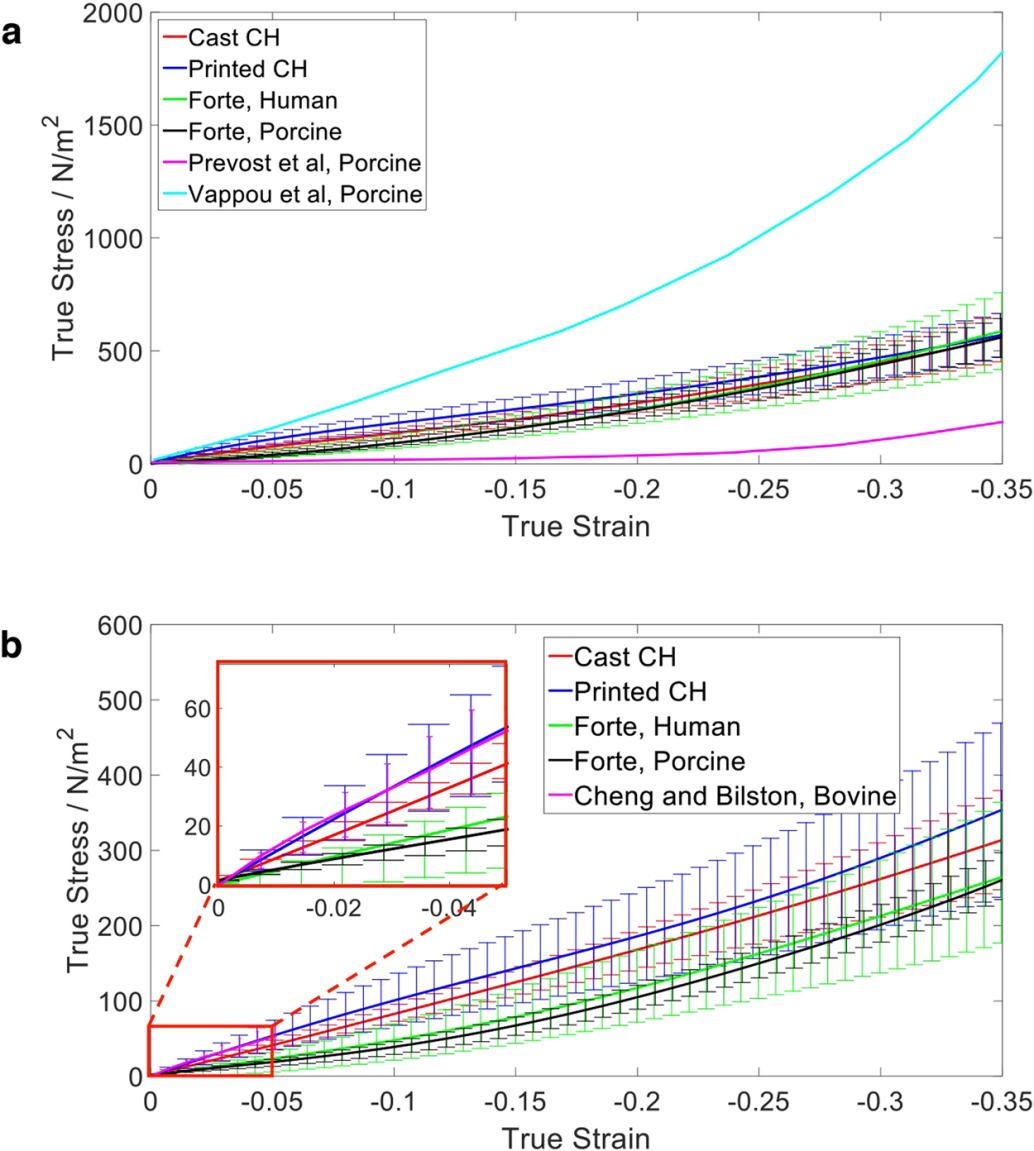
Stress-strain curves of printed and cast 5 wt% PVA 0.59 wt% Phytagel at (**a**) 0.01 s^−1^ and (**b**) 0.0001 s^−1^ strain rate^26–29^.

The results from the compression tests of the printed samples compared to the cast samples are shown in Fig. 1a. The stress-strain curves show a good agreement between the mechanical properties of the samples produced by the two different methods. The average true stress at 30% engineering strain at 0.01 and 0.0001 s^−1^ strain rates for cast moulded and printed samples are summarised in Table 1. The average stiffness of the printed samples was greater than that of the cast samples for both strain rates; however, the findings are well within one standard deviation of each other. The viscoelastic nature of the composite hydrogel is also exhibited by the 3D printed method, as the stiffness is greater at higher strain rates, demonstrating strain rate dependency.

**Table 1:** True stress at 30% engineering strain.

Fabrication Method	True Stress (Pa) ± Standard Deviation	
	0.01 s^−1^	0.0001 s^−1^
Cast Moulded	577.75 ± 111.12	321.34 ± 68.53
3D Cryogenic Printed	585.49 ± 97.33	363.10 ± 119.23

In addition, a comparison between the mechanical response of the printed composite hydrogel and real brains tested in literature at the same strain rates is shown in Fig. 1b. The results for 0.01 s^−1^ strain rate show that the printed CH is within the wide range of real brain results obtained from Forte *et al*.^26^, Prevost *et al*.^27^ and Vappou *et al*.^28^. For the 0.0001 s^−1^ strain rate, the stress-strain curve has a wider error range than those previously reported by Forte *et al*.^26^, but still well within the range of brain tissues results reported in the literature, and especially comparable to the results at 5% strain reported by Cheng and Bilston *et al*.^29^.

### Scanning electron microscopy images

**Figure 2:**
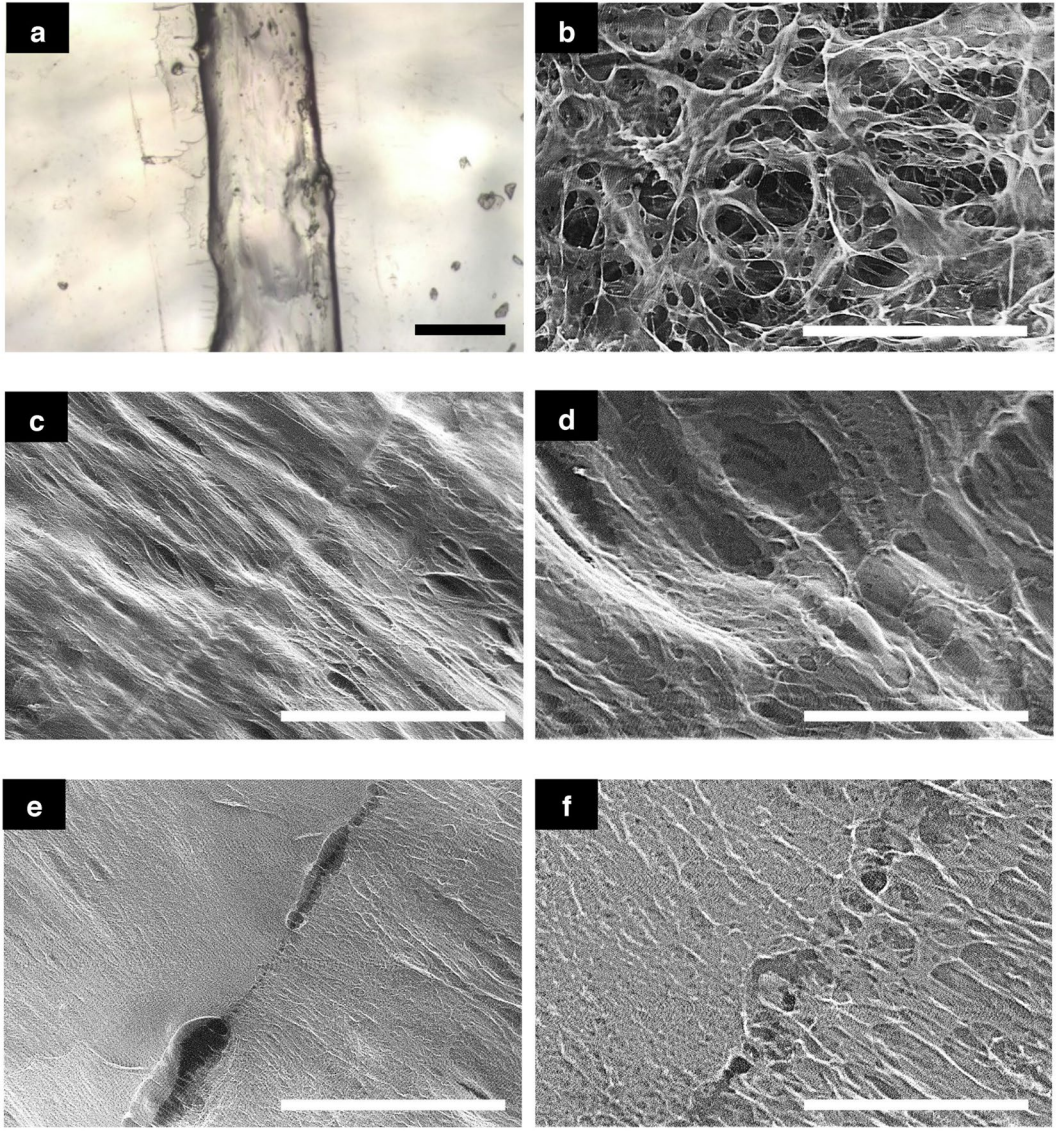
(**a**) White light interferometer image showing precision of printed material; SEM images of (**b**) general microstructure, (**c,d**) crosslinking between layers, (**e,f**) flaws between layers. Scale bars, (**a**) 200 µm, (**b**) 100 µm, (**c**) 500 µm, (**d**) 100 µm, (**e**) 500 µm, (**f**) 100 µm.

SEM on 6 samples that underwent one freeze-thaw cycle were conducted to reveal the morphology of the CH microstructure. Images are shown in Fig. 2. The general microstructure of the material was homogeneous, with a pore size of approximately 10–20 µm, as shown in Fig. 2b. The scans also revealed the branching morphology of the 3D printed material microstructure.

SEM analysis was also conducted on the connecting regions between consecutive printed layers and these are shown in the two sets of images in Fig. 2c,d,e,f . Figure 2c,d clearly demonstrates the physical cross-linking across different layers. The pore size is consistent with what has been previously shown^23^. Figure 2e,f reveals bubbles of ~100 µm diameter trapped between layers due to imperfections in the surface of the previous layer.

### Printed complex structures

**Figure 3:**
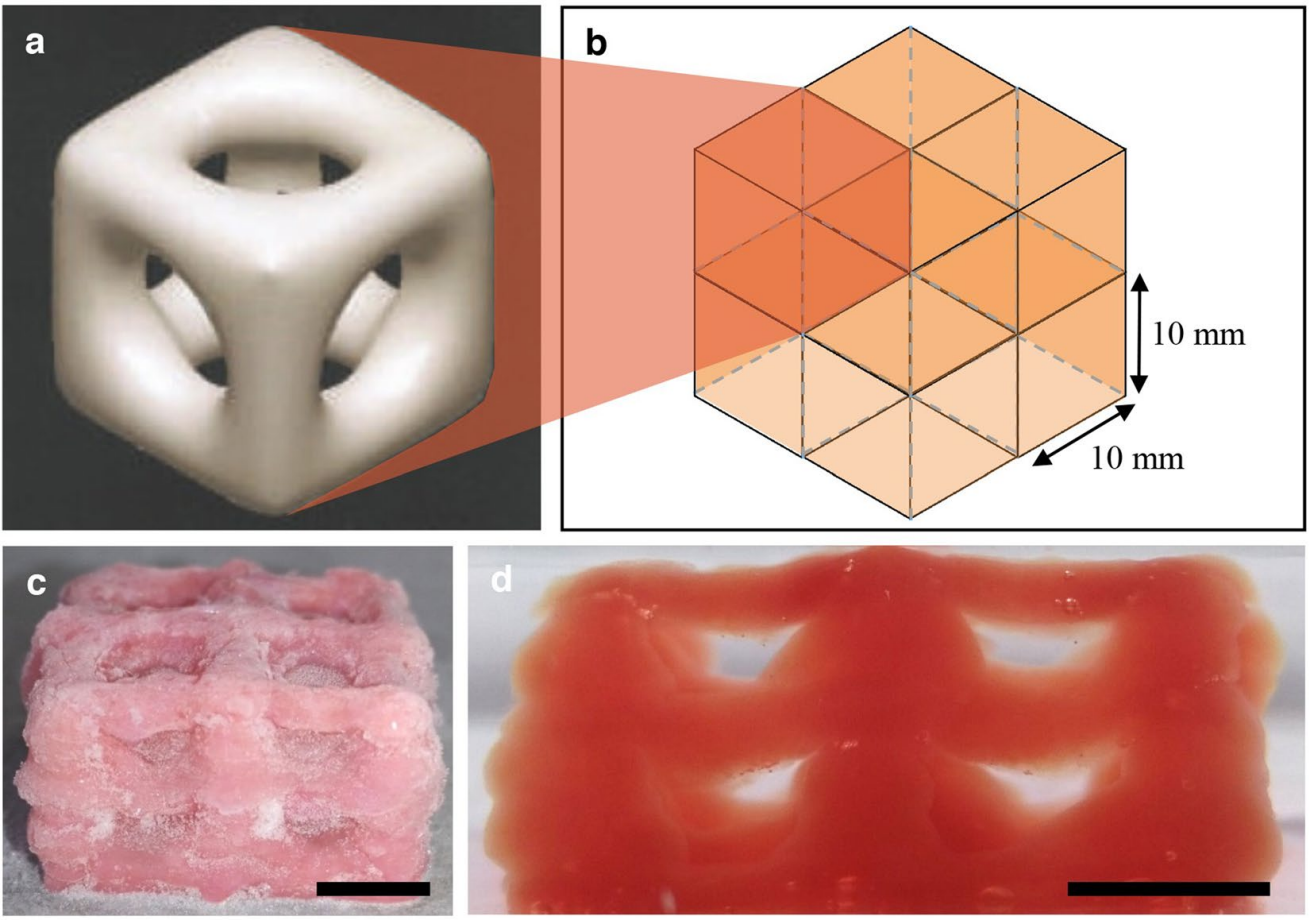
(**a**) Cylindrical pore microstructure, adapted from^47^ and (**b**) 8 unit cells printed; thawed printed 8 cell structure in (**c**) isometric view and (**d**) side view. Scale bars, (**c**) 10 mm and (**d**) 5 mm.

Figure 3c,d show the hollow printed structures. A structure of 10 mm height featuring two layers of cubic hollow geometry as shown by the schematic in Fig. 3a was successfully printed using 5 wt% PVA 0.59 wt% Phytagel composite hydrogel ink. Figure 3c and d shows the printed structure in the frozen state and the thawed state respectively. Upon thaw, the voids in the centre of each unit cell are able to retain water and therefore, the structure remains hydrated for a longer period of time. The presence of frozen water trapped inside the central void of the cubic structure also structurally supports the hydrogel pillars. The pillars of the unit cells were 3 mm in diameter so the printed structure was still prone to collapsing under gravity once thawed. Nonetheless, the structure appeared uniform and homogenous throughout and did not exhibit any failure due to poor crosslinking. The distinction between liquid water and cross-linked hydrogel can be seen clearly in Fig. 3c. The images shown in Fig. 3c,d revealed the printing method was able to successfully produce the desired multi-layered complex structure with super soft bulk stiffness.

### Cell viability on soft hydrogels

**Figure 4:**
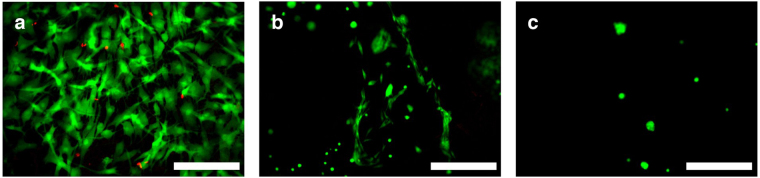
Cell viability on (**a**) collagen, (**b**) poly-l-lysine and (**c**) gelatin coated 3D printed CH. All scale bars are 100 µm.

Figure 4 shows cell viability on 3D printed CH previously coated with collagen gel (a), poly-L-lysine (b) and gelatine (c), after 72 hours in culture. Live cells were more than 97% on collagen-coated CH, confirming a good biocompatibility of the materials. Collagen gel coating showed the best results in terms of cell attachment and viability. Compared to the collagen coating, live cells attachment on the poly-L-lysine and on the gelatine coated hydrogel were respectively, 76% and 8%. Cell viability was greater than 98% on both the poly-L-lysine and gelatine coating.

Cell morphology was different for each coating. Cells on the gelatine-coated substrate attained a more rounded morphology. 80% of the live cells observed were well-spread on the poly-L-lysine coated hydrogel, and all cells were well-spread on the collagen-coated materials. Although the cell viability and spreading were similar on both poly-L-lysine and collagen coatings, there were more distinct regions of tightly packed cell clusters observed on the poly-L-lysine coated hydrogel. This indicated that cell attachment was less homogeneous, and the cells favoured a more densely packed configuration than on the collagen coated hydrogel. Cell attachment for poly-L-lysine and gelatine coated 3D printed CH has been achieved with optimized coating processes, which are currently under further investigation.

## Discussion

A composite hydrogel, of a composition that mimics soft tissues, was used as the ink material for cryogenic 3D printing. The use of dry ice, which is a safer alternative to liquid nitrogen, in an isopropanol bath to create the cryogenic stage was adequate at providing a sustained thermal sink, allowing the ink to freeze on contact with the stainless steel plate and preceding layers. The thermal energy required to freeze the deposited ink was negligible compared to the rate of energy removed by the dry ice, so new layers were not able to raise the temperature of the plate to the point of melting the material. Up to ~20 mm printing height, the samples were able to freeze upon contact with the prior layer. As the height increased, the time taken for a layer to freeze completely increased, which was taken into account when designing the code for the printer in terms of ink flow rate and speed.

The printing of the solid cylindrical samples were very repeatable and failed prints were not common, leading to a printing success rate of 95%. In the remaining 5%, the print failed for obstruction of the nozzle which impeded the deposition of the first layer of material. More repeats were executed to obtain a good print for the 2 layered 3D structure, due to the complex 3D geometry. This was mainly due to imperfections in the printing of each layer. The unevenness was then amplified in subsequent layers as the ink flow was caught on any protuberance in the preceding layer. When plastics are used as ink in commercial 3D printing, the printer is able to smooth down any protrusions in the previous layer to remove this issue. However, it was difficult to apply the same smoothing technique with the cryogenic technique presented in this article as the hydrogel froze immediately when deposited. Once the build-up of material at any point on the printed structure reached a critical level, the print failed. Evidence of this may be found in the Supplementary Information (S1). In future, the print quality consistency can be improved by ensuring an extremely constant ink extrusion flow rate and limiting the inaccuracies caused by condensation through improved environmental control.

The 3D printed scaffolds demonstrated the concept of using a separate material to support the printed hydrogel to be able to create structures that would be extremely difficult to cast-mould. The support material was water, chosen for its similar freezing point to CH and biocompatibility. The water support did not affect the cross-linking of the hydrogel and, upon thaw, fully intact 3D structures were left once the supporting water had drained away. Owing to the nature of water, no harsh chemicals are required to wash the supporting material away. Therefore, this simple and biocompatible technique highlights the potential of this method to be used in the fabrication of very soft complex 3D scaffolds, which could be exploited for tissue engineering purposes.

The low viscosity of the supporting water resulted in a rapid spreading of the liquid before solidification. This reduced the precision of the deposited water and therefore, the use of other materials with tuneable viscosity may improve the quality and should be investigated in future work. A candidate for a support material would be gelatine as it is biocompatible, has tuneable viscosity, inexpensive and removable when heated to a cell-appropriate temperature of 37 °C, as Hinton *et al*.^15^ have shown in their work. An advancement of the cryogenic stage would be to build a setup that allows precise control of the temperature of the plate so that the material may be deposited at a temperature that allows the ice crystals to form slowly so that the nozzle is able to smoothen out the deposited layer before continuing to print further layers on top. Once this has been achieved, the temperature of the plate should be controlled so that only the previous layer is frozen, whilst leaving future deposited material semi-solid. Consequently, the temperature of the plate should be decreased as the printing height increases.

The ink used in this work is a composite hydrogel of poly(vinyl) alcohol (PVA) and Phytagel, which has been pioneered by Forte *et al*.^23^ to mimic soft tissues with stiffness of O(1) kPa. This polymer blend is also able to reproduce the relaxation response of brain over other mimicking materials (such as agar gel, gelatine and polyacrylamide (PA))^22,30^. PVA hydrogel requires a freeze-thaw cycle to form physical hydrogen bonds that crosslink the material matrix. Therefore, this ink synergises with the cryogenic method and provides a simple solution that incorporates the crosslinking of the composite hydrogel in a single step.

Mechanical compression analysis of the stress-strain relationship between the solid samples of printed and cast 5 w% PVA and 0.59 wt% Phytagel showed good agreement, which indicates cryogenic printing is an effective method for the fabrication of super soft hydrogels. The average stress at 30% strain for the printed samples were greater than the cast molded samples by 1.3% and 13.0% at 0.01 and 0.0001 s^−1^ strain rates, respectively. This result may be due to a double thawing process as the 3D printed samples experienced 1 minute of thawing as they were transported from the printing platform to the storage freezer. During this time, a small degree of crosslinking may have formed, which would contribute to the slight increase in stiffness of the 3D printed samples compared to the cast moulded samples. However, the compression results are within one standard deviation of the average stress.

Furthermore, the results for the stress-strain curves of the printed samples showed no evidence of fracture between layers, although shear tests should be performed in future to further validate this. The 3D printed constructs exhibited a larger standard deviation from the mean curve, which could indicate that the micro bubbles formed between layers caused by this method have some influence on the mechanical behaviour of the material. The findings demonstrate that the printed material is very well matched to the work published by Leibinger *et al*.^22^ and Andrikakou *et al*.^18^ and hence well matched to very soft tissues, such as brain and lung. Furthermore, the printed material also matches the mechanical response of brain at two different strain rates, hence exhibiting viscoelastic behaviour. By using the cryogenic method, we are now able to 3D print super soft viscoelastic complex structures with a stiffness of O(1) Pa to a precision of O(100) µm, which is an unprecedented advancement in the capabilities of printing soft hydrogels.

It is widely known that the histology of real soft tissues, in particular brain, is complex, which makes it difficult to test due to its sensitivity to environmental testing conditions and post-mortem time, among other factors. For both strain rates, the stiffness response of the 3D printed composite hydrogel is within the range of real brain results reported by other authors^26–29^. Furthermore, in comparison to real brain, the printed CH offers a greater repeatability and efficiency.

Another factor which may contribute to the slight increase in maximum stress at 30% strain for the 3D printed material compared to the cast moulded samples is the difference in freezing rate between two different methods. For the 3D printed samples, the freezing rate also decreases slightly as the layer height increases. However, it has been shown in literature that the freezing rate used when fabricating other PVA composite cryogels does not affect the mechanical properties of the hydrogel as greatly as the thawing rate, which is where the majority of the physical cross-linking bonds are formed^31–35^. Additionally, evidence that supports this is shown in Fig. 2(c)–(f) and in the Supplementary Information (S2), where a consistent average pore size across all layers is not greatly affected by the slight changes in freezing rate. A further study on how the thawing rate affects the stiffness of the CH can be found in the Supplementary Information (S3).

Structures of greater stiffness that can mimic other human soft tissues can also be achieved using the CH as ink. CH offers stiffness tunability by varying the composition of the constituent hydrogels. However, the viscosity of the ink increases when the concentration increases. The viscosity is a critical factor for this pneumatic extrusion based printing method. In future, additive silk particles, which have been used to greatly strengthen PVA without hugely affecting the solution viscosity, should be explored to create stiffer structures.

From the SEM images, the homogenous microstructure of the 3D printed solid structures is well matched with the scans obtained by Forte *et al*. of cast-moulded CH in terms of pore size and morphology. This indicates that physical cross-linking in the composite material does not occur during the freezing stage since the layers undergo the phase change at different times but instead crosslink during the thawing stage, which is consistent with our knowledge of cryogel physical bonding kinetics.

Additionally, images taken of the region across consecutive layers exhibit a clear crosslinking hydrogel network with similar porosity and morphology to the rest of the microstructure at 100 µm scale. This suggests that the hydrogel is able to bond across layers even though the fault line is clearly distinguishable at a wider view of 500 µm. Due to minor inaccuracies in the deposition of the hydrogel ink, micro bubbles of around 100 µm in size are formed, which have been captured in the SEM images. The size of these defects compared to a layer height of 1 mm indicate that the bubbles should not affect the response of the structure on the macroscopic scale, and indeed, the stress-strain curves from the compression tests show that these faults have little effect on the value of stress obtained at 30% strain compared to cast-moulded samples. This, however, might affect the resistance to damage of the printed material, which will be investigated in future studies. Interestingly, the hydrogel network exhibits diffusive behaviour in an apparent effort to mend those tears by crosslinking across the gap. Where the voids are small enough, the hydrogel can be seen to be able to cross-link and branch across the void (Fig. 2 f). This is a very interesting observation as it suggests that the hydrogel is able to diffuse through the ice as it thaws whilst forming the network structure. This may suggest a self-healing mechanism as it allows the material to amend the errors created by an inaccurate print to some degree.

Cell viability studies on collagen and poly-L-lysine coated CH substrates were carried out to assess the potential of the composite hydrogel and printing method for future use in a wide range of bioengineering applications. Additionally, gelatine coatings were studied, as it is a more affordable alternative to collagen. Cell viability and attachment were excellent on collagen-coated hydrogels. This was expected considering that collagen is widely known as a favourable material on which cells thrive since it provides them with sustenance to grow^36^. Compared to the collagen coating, cell attachment was lower by 26% on poly-L-lysine coated CH. Only rounded cells were found on gelatine-coated samples.

Furthermore, the poly-L-lysine coating is a very promising alternative as it functionalises the hydrogel through adsorption providing a more robust and durable coating. Cells exhibited a good attachment, although the morphology was slightly inconsistent, with large spreading of some cells and low spreading of others. Small regions of tightly clustered cells were observed, creating localised areas with high cell attachment. Despite this, the poly-L-lysine coating has demonstrated great potential as a coating that greatly enhances the cell attachment of the substrate. It is expected that advancing the adsorption process will improve the homogeneity of the coating and therefore enhance cell attachment, comparable with collagen-coated hydrogels. Future work will focus on improving the adsorption of poly-L-lysine onto the hydrogel, which may be done by using a solution of pH 11 and radiating under UV^37–39^.

The gelatine coating was unable to provide substantial results due to the thermal reversibility of gelatine at the incubation temperature of 37 °C, where the coating, which was a gel at room temperature, turned into the liquid state. Nevertheless, further work into the thermal stabilisation of gelatine using UV radiation will be considered in future studies.

A full comparative study involving an in-depth analysis of coating efficiency has not been performed in the current work since was not in the scope of this investigation. This is a limitation of the present study and will be carried out as a follow-up investigation in the near future.

Dermal fibroblasts were used to evaluate the biocompatibility of the printed constructs. This was a deliberate choice as the cellular evaluation was an initial screen to determine the general biocompatibility of the newly designed scaffold, rather than a specific biofunctional study. Furthermore, considering the wide range of applications of these super soft hydrogels, with great potential for closely mimicking various soft tissues, fibroblasts were chosen since they showed to adapt to various material stiffnes^40^. Moreover, it has been shown by Volckaert and De Langhe that fibroblasts are involved in lung development, and fibroblasts growth factors (FGFs) pathways are crucial for the regulatory fibroblast-epithelial cell cross-talk^41^.

Collagen has been commonly used as biomaterial for 3D scaffolds in neural tissue engineering, thanks to its versatility, biocompatibility, low antigenicity, inflammatory and cytotoxic response^42^.

It has also been shown that neuronal cells are able to survive for more than 42 days and maintained high cell viability in collagen scaffolds^43^.

All these evidences pave the way for future in-depth biofunctional studies of our 3D printed scaffolds coated with collagen in combination with various cells types, including neural cells, to determine a more functional ‘tissue-like’ response of the material, mimicking natural soft organic tissues.

In summary, a printing technique focusing on producing soft structures using a composite hydrogel as ink has been developed. The current efforts are focused on fabricating complex structures for a wide range of tissue engineering and mechanobiological applications that are extremely difficult to achieve using a cast-moulding method because of their super soft characteristics and hollow geometries. This was accomplished by incorporating a cryogenic procedure. The use of a freeze-plate allowed the CH ink to instantly solidify upon contact with the plate. Each layer was built on previous solid layers and a stable 3D structure was created. This technique integrates the freeze-thaw process required for the formation of physical hydrogen bonds between the –OH groups of the PVA and Phytagel and is therefore and elegant and time-reducing method, owing to the instantaneous freezing step. The crucial factor in achieving successful prints is to ensure the evenness of each printed layer, which should be a key area for development in the future.

Upon thaw, the material properties of the CH were very closely matched to that of the traditional cast-mould fabrication method, and well within the standard deviation of the samples tested. Moreover, SEM scans revealed that the microstructure of the printed CH was well matched to previous findings of cast-moulded CH in terms of pore size and morphology, further validating this method. The physical cross-linked network was exhibited across different printed layers and formation of bonds across boundary defects, such as voids, suggests a self-healing mechanism during the thawing stage.

The use of the CH as an ink in cyrogenic 3D printing opens the doors to many applications that have yet to be explored due to the inability to fabricate super soft materials that mimic the stiffness of brain, lung and other human tissues characterised by a complex geometrical structure. This article has shown that the cryogenic technique used for the fabrication by 3D printing of the CH provides the capability to manufacture synthetic materials of complex shape and good mechanical properties and microstructure. The excellent biocompatibility of the CH with a collagen coating will allow the hydrogel structures to be used in various mechanobiology and, potentially, regenerative medicine studies of soft tissue-like CH substrates, including the future exploration of neuronal cell seeding. In addition, the CH may also be used as a mechanically accurate tissue phantom for surgical training and also for more in-depth and destructive tests that are unethical to perform *in vivo*, such as impact experiments to study *e*.*g*. traumatic brain injuries.

## Materials and Methods

### Composite hydrogel ink preparation

PVA (molecular weight 146,000–186,000 Da), Phytagel and deionised water were supplied by Sigma Aldrich, USA. A concentration of 2.5 wt% PVA and 0.295 wt% Phytagel (obtained by dissolving 5 wt% PVA and 0.59 wt% Phytagel powder separately, see below) was used for the ink as this is the composition used by Leibinger and co-workers to match the stiffness of porcine brain up to 95% strain^22^. A detailed descriptions of the constituent hydrogels is provided in the previous work by Forte *et al*.^23^.

The CH ink solution was prepared by dissolving 5 wt% PVA and 0.59 wt% Phytagel powder separately in deionised water for 1 h at 90 °C^23^. The separate solutions were then combined together in a 1:1 weight ratio and kept at 70 °C under constant stirring for an additional 30 min. The mixed solution was then allowed to cool to room temperature to be used as ink for the printing process, where it remained in liquid phase.

### Cast-moulded samples

For mechanical comparison with the 3D printed samples, control samples were prepared using a standard cast-moulding technique. The CH solution, which was prepared from the same batch as the printing ink, was transferred into a plastic mould and frozen at −25 °C for 15 hours. The material was then thawed at room temperature and tested. Samples of 10.65 ± 0.16 mm diameter and 6.39 ± 0.26 mm height were cut using a biopsy punch.

### 3D printing process

A commercial 3D printer, Ultimaker^2^ (Ultimaker, Netherlands), was modified for the purposes of 3D printing soft structures. The 5 wt% PVA 0.59 wt% Phytagel solution does not solidify at room temperature, thus allowing the ink to be extruded.

**Figure 5:**
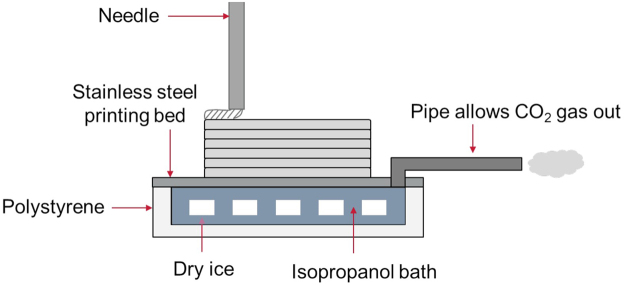
Schematic of the cryogenic 3D printing procedure and set up (not to scale).

An extrusion based printing method was developed using needle of gauge 21 (0.514 mm internal diameter) connected to a perfusor (B. Braun, Germany) by PTFE tubing. A stainless steel plate, chosen for its good thermal conductivity of 12.44 W/(mK)^44^, was kept in contact with dry ice, which has a sublimation temperature of −78.5 °C. The contact was kept constant across the plate using isopropanol, with a melting point of −89 °C, as a thermal conductive fluid with thermal conductivity of 0.14 W/(mK) at 21 °C and atmospheric pressure^45^, which therefore remained in liquid phase when in direct contact with the dry ice pellets. The dry ice and isopropanol bath were contained in an insulating polystyrene container, with thermal conductivity of 0.03 W/(mK)^46^, which had an outlet to vent the sublimated CO_2_. Due to the high water content of the hydrogel, the solution froze immediately upon contact with the conductive stainless steel plate. This solid state allowed a stable structure to be built by means of a layer-by-layer approach. A schematic demonstrating the setup of this cryogenic printing method is shown in Fig. 5.

### Printed cylindrical structures

Using the CH ink solution, cylindrical samples were printed to match the same dimensions as the cast-moulded samples. The print settings are shown in Table 2. The printing path was obtained by importing a stl file made in Solidworks (Dassault Systemes, France) into the 3D printer software (Cura, Ultimaker, Netherlands) in order to generate the toolpath (gcode file), readable by the 3D printer. Following the completion of the print, the printed samples were immediately stored at −25 °C for 15 hours, to be as consistent as possible with the conditions of the cast samples. Before testing, the samples were thawed at the same rate as the cast-moulded samples since it has been shown that the mechanical characteristics of the PVA component depend highly on the thawing rate, as the slower the rate the more time is allowed for a physical network to form^31^. The dimensions of the final printed samples were 10.25 ± 0.1 mm diameter and 6.75. ± 0.13 mm height (reported as mean and standard deviation), which show percent deviation below 1% and 2% respectively for this particular geometry. Untested samples were refrozen to analyse their microstructure under SEM.

**Table 2:** Structural dimensions and corresponding printer settings for solid cylindrical samples.

Diameter (mm)	Height (mm)	Ink Width (mm)	Print Speed (mm/s)	Print Flow Rate (ml/h)
10	6.5	1	5	6

### Printed complex structures

To evaluate our cryogenic 3D printing method for the fabrication of soft tissue scaffold constructs and to demonstrate its precision, structures with complex geometries were printed. Therefore this method also demonstrates the viability to print these complex structures that would be difficult to fabricate by cast-moulding and can be used as cell scaffolds for future mechanobiological studies.

The hollow structures were printed based on a cylindrical pore scaffold microstructure, shown in Fig. 3a, suggested by Hollister *et al*.^47^ in order to have the porosity and stiffness required for cells to successfully thrive. A schematic of the 3D structure to be printed is shown in Fig. 3b. These structures were achieved by introducing deionised water as a support material. At the beginning of the process, the CH ink was deposited using the same cryogenic setup described previously. After the completion of each layer, the supporting liquid water was deposited layer-by-layer using a needle and syringe guided by hand. The liquid water changed phase to solid ice when it contacted the printing plate and frozen material from previous layers, thus providing a stable surface. This allowed subsequent layers of CH ink to be deposited on the supporting solid ice layer. Upon print completion, the structure was removed from the plate and allowed to thaw to room temperature. The supporting ice melted away, leaving the hydrogel network intact.

Complex 2D geometrical structures were also printed and are shown in the Supplementary Information.

### Mechanical characterisation

Unconfined, uniaxial compression tests, up to 30% strain, at 0.01 and 0.0001 s^−1^ strain rates were carried out on both cast and printed cylindrical solid samples (*n* = 6). A Mach-1^TM^ mechanical testing system (Biomomentum, Canada) with a 1.5 N load cell (Honeywell, USA) was used to carry out the tests as it is designed towards soft material studies. A 100 Hz sampling rate was used and the data was filtered using a software integrated low-pass filter of order 2 and cut-off frequency 50 Hz. Samples were kept well hydrated prior to testing and immediately transferred onto the machine platen. Each test took 30 s, which is a sufficiently short time to confidently rule out dehydration effects on the samples. Stress-strain curves were plotted and compared for the cast and printed samples to show that the cryogenic technique produces a mechanically similar material compared with the cast-moulded technique.

The compressive stiffness was characterised by the stress-strain curves calculated from the axial force and displacement data obtained from the experimental tests. The diameter and initial height of each sample was recorded in order to calculate the true stress and strain. Given the very large water content, incompressibility was assumed for the CH in the calculations; this is also conventionally used when modelling real brain, which the CH has been shown to match in compression up to 30% strain^23,48^.

### Analysis of printed microstructure morphology

SEM analyses of various features of the printed material microstructure and surface morphology were conducted for further validating the cryogenic printing method. The cross-section of the 3D printed structures were prepared for microscopy using the freeze-fracture method. The surface was then gold-sputtered using the Auto Sputter Coater (Agar, UK) to allow for the conduction of the electron beam and to help preserve the microstructure. An environmental SEM (ESEM) 15 kV laser beam was used with 50 pA in a variable pressure mode of 100 Pa at a working distance of 10 mm. The regions investigated were the internal homogenous microstructure, the bonding between consecutive layers and the sample surface with the intention of validating the cross-linking of the hydrogel throughout the entire printed material.

### Cell seeding

Primary Normal Human Dermal Fibroblasts (NHDF, Promocell, Germany) were seeded onto the 3D printed 5 wt% PVA 0.59 wt% Phytagel hydrogels, at a density of 4 × 10^4^ cell/cm^2^. Samples were previously coated with collagen, poly-L-lysine or gelatine to enhance cell attachment. Collagen has been used as a coating for cell attachment by Engler *et al*.^49^ to study the effect of substrate stiffness on cell differentiation, which therefore indicates that the cells have the ability to feel the underlying substrate stiffness through the collagen coating. Gelatine is often used as an alternative tissue scaffold material to collagen due to its greater availability and cost effectiveness. Poly-L-lysine has also been used as a coating as it forms a cationic layer that attracts the anionic sites on the cells surfaces^37,50–52^. Whereas the collagen and gelatine coating rely on absorption into the pores of the hydrogel where they crosslink and attach to the substrate, the poly-L-lysine is electrostatically adsorbed onto the surfaces owing to the –OH functional groups present in the PVA and Phytagel chains. This creates a stronger bond that cannot be physically removed.

### Cell viability

Cell viability was assessed using Live/Dead staining. Samples were kept in culture for 72 hours and then stained using calcein for live cells and ethidium homodimer-1 (EthD-1) for dead cells. Samples were immersed in a phosphate buffered saline (PBS) solution, containing 2 μl of 4 mM Calcein AM (acetoxymethyl) and 1 μl of 2 mM EthD-1 for each ml of solution, and then incubated for 15 minutes at 37 °C. Live (green, FITC) and dead (red, TRITC) cells were then observed under a fluorescence microscope.

## Electronic supplementary material

Supplementary Information
