# Supplementary material for: Cryogenic 3D Printing of Super Soft Hydrogels
Source: Sci Rep. 2017 Nov 24;7:16293. doi: 10.1038/s41598-017-16668-9 (PMC5701203; doi:10.1038/s41598-017-16668-9)
Supplement: Supplementary file 1 — Supplementary Information [file 41598_2017_16668_MOESM1_ESM.pdf]

Cryogenic 3D Printing of Super Soft Hydrogels

Supplementary Information

Zhengchu Tan[1], Cristian Parisi[2], Lucy Di Silvio[2], Daniele Dini[1] and Antonio E. Forte[3]*

[1]*Department of Mechanical Engineering, Imperial College London, South Kensington Campus, Exhibition Road, London SW7 2AZ*

[2]*Tissue Engineering and Biophotonics Division, King's College London, Guy's Hospital, Great Maze Pond, London SE1 9RT*

[3]*Department of Bioengineering, Imperial College London, South Kensington Campus, Exhibition Road, London SW7 2AZ*

**Corresponding Authors:* antonio.forte10@imperial.ac.uk

S1. 2D Printed Complex Structures

The print settings were optimised for the printed structures. The settings that produced the most stable results are shown in Table 3. The toolpaths for these structures were generated using a proprietary MATLAB script (Mathworks, USA).

Table 3. Structure dimensions and corresponding printer settings for complex structures.

**Unit Cell Dimensions (mm)**	**Ink Width (mm)**	**Print Speed (mm/s)**	**Print Flow Rate (ml/h)**
5x5	0.5	5	6

The results for the printing are shown in Fig. S1d-i. The settings used to produce these structures gave the most consistent and stable prints. The results show that the hexagonal pattern gives the smoothest print. This is because the angles in the corners are wider so less material is built up. Therefore, precision depends on printed geometry as well other factors such as the flow rate and print speed. Multiple layers were tried for all three patterns. The highest number of stable, smooth layers was achieved by the hexagonal structure and the height was recorded at approximately 3-4 mm.

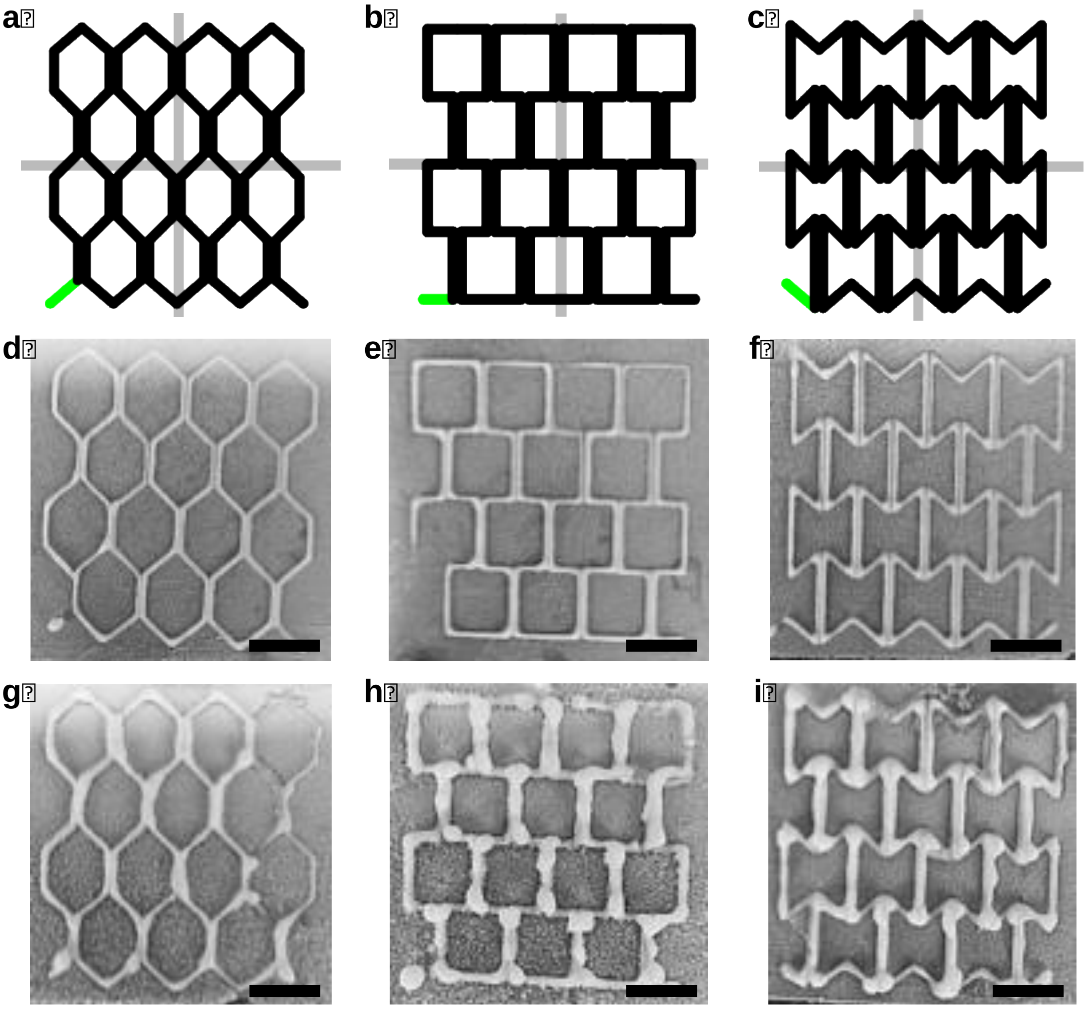

Figure S1: Gcode visualisation of complex printed structures (a) hexagonal, (b) orthogonal, (c) re-entrant auxetic; first layer prints of (d) hexagonal, (e) orthogonal and (f) re-entrant auxetic; 3 layer prints of (g) hexagonal, (h) orthogonal and (i) re-entrant auxetic structure. All scale bars are 5 mm.

Three different patterns; re-entrant, orthogonal and hexagonal, were chosen to investigate the effect of printing angles on the quality of the printed structures as they feature acute, at 90° and obtuse angles respectively, as shown in Fig. S1a-c. Additionally, the re-entrant structure is auxetic, meaning it has a negative Poisson’s ratio, which is a fascinating mechanical attribute that has also been found in specific parts of the body such as skin and bone[51,52].

The results in Fig. S1d-e show that the geometry of the printed structure affects the quality of the print. The use of a constant printing speed and flow rate results in the build-up of material at certain regions of the geometry, such as the corners. The number of layers achieved before print failure due to the build-up of imperfections in the printed surface was increasing in the following order: auxetic, orthogonal and hexagonal honeycomb as is shown in Fig. S1g-i. In future, the MATLAB code should be further developed to increase the speed of the printing head when going round a corner as a function of the angle, faster speed for sharper angles, to achieve smoother layers.

S2. Freezing Rate

In addition to Fig. 2 (c)-(d), the SEM images in Fig. S2 show that the pore size is consistent throughout all layers of the cryogenically printed sample, at ~20 µm. This suggests that the freezing rate, which decreases slightly as layer height increases, has little effect on pore size. Fig. S2 shows two different magnifications of the layer connection between layers 3 and 4 (scale bar of 1mm and 100 µm respectively).

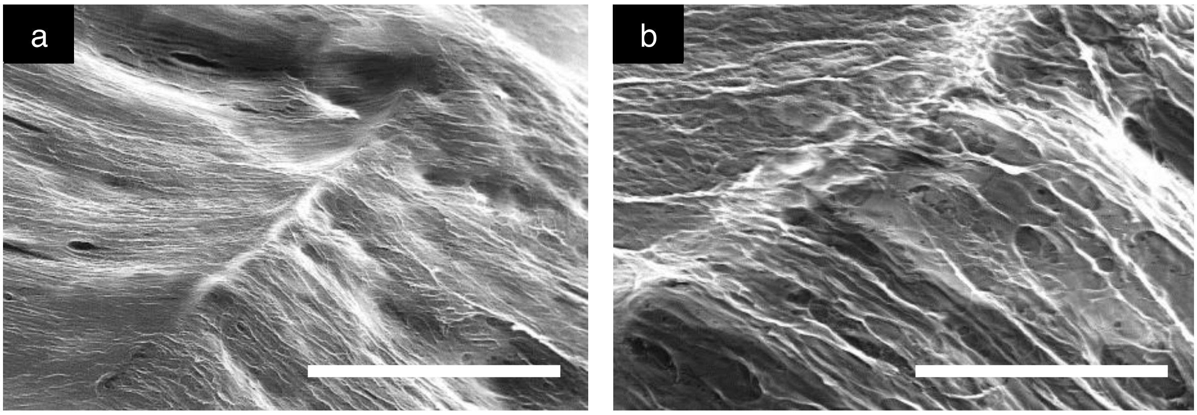

Figure S2: SEM images of layer connection: layer 3 on the left and layer 4 on the right, in different magnification. Scale bars, (a) 1 mm, (b) 100 µm.

S3. Thawing Rate

There is also evidence to confirm that the thawing rate plays a large role in the cross-linking behaviour and strength of the physical bonds. For samples with a higher thaw rate of 2.13 °C/min compared to 0.27 °C/min, the bonding between layers was weaker and layers were observed to separate from each other. In addition, the mechanical response of samples thawed at these two different rates was recorded and the result show that the higher thawing rate results in a significantly lower mechanical stiffness. This suggests that the physical bonds between layers requires more time to form and strengthen than in a single layer. Moreover, given enough time, chains are able to form across the condensation between layers formed during the 3D printing process.

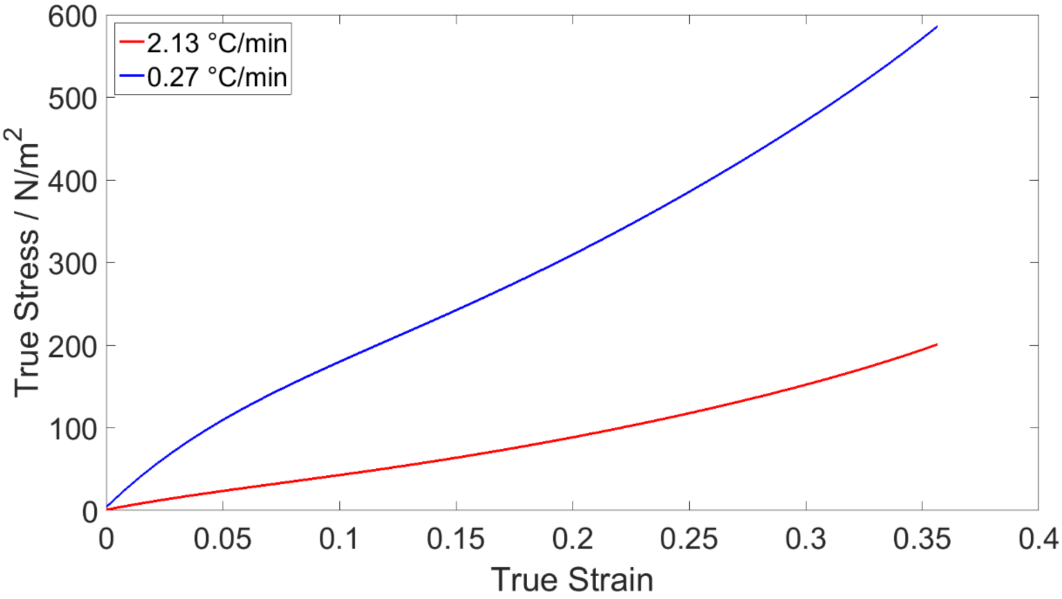

Figure S3: Mechanical response of 5 wt% PVA 0.59 wt% phytagel at 2 different thawing rates.
